# 

**Published:** 1849-06

**Authors:** 


### INDEX TO VOL. V.

PAGE.

Anæsthesia, by Dr. Griscom, 42
" by Prof. Simpson, 114
" in midwifery, 590
Anæsthetics, new, by Thos. Nunnelly, 46
" new candidate for the discovery of, 501
American Medical Association, (editorial,) 49, 690
" " " committees, 430
" " " transactions, 612
" " " and Medical schools, 370
Annalist, 179
Association, Southern central N. Y. 179, 628
" Buffalo Medical, report of committee on compensation for public medical services, 435
Asphyxia neona torum, 227
Aneurism axillary, case of, by J. B. Flint, M.D. 410
Aneurism cured by compression, case of, 361
Abortion, procuring, 496
Auscultation, intestinal, 295
Amputations, Dr. Sedillot on, 561
Alopecia, case of, 339
Ayre on Cholera, 342, 357
Aconite, therapeutic effects of, 737
Bryan, Prof. James, translation of Jules Roux on annnihilating pain in surgical operations, 1
" " case of gun shot wound in abdomen, 6
Bright's disease a blood disease, by Prof. Walshe, 605
Bowlegs, 608
Burwell, Dr. Geo. N., on chloroform in midwifery, cases, 8, 441
Brigham, Dr., death of, 375
" Amariah, obituary, 397
Butterfield, John, Prof., decease of, 375
Buffalo Med. college and N.Y. Annalist, 55
" " " 244, 429, 376
" " " commencement, 568, 629
Brain, Hydatids in ventricles of, 80
Bismuth, large doses of, in gastro-intestinal affections, 298
Bowman's practical chemistry, 118
Blackwell, Miss, M.D. 175, 414
Barth and Roger, manual of auscultation, 176
Bladder, chronic inflammation of, injections of nitrate of silver in, 228
Bowditch, Dr., on malignant tumor, 419
Boutigny, P. N., incombustibility of man, 470

PAGE.

Bougie, extroduction of, by Prof. Brainard, 338
Blake's experiments to determine the quantity of blood in the body, 356
Brainard on vaginal ulcerations, 729
Chloroform in midwifery, Dr. Geo. N. Burwell, 8, 441
Chloroform liniment, 150
" death from, 681
Collodion blistering, 362
Cryptogamic origin of fevers, by Prof. J. K. Mitchell, 60
Cold Water in Scarlatina, 363
Cholera, progress of, 64
" and homœopathy, 99
" treatment of at Louisville Ky., 100
" report on diagnosis of, by A. Flint, M.D., chairman of committee, 121
" treatment of, by Dr. Shanks, 155
" fluid secretion in, by M. Mialhe, 171
" by M. Ward, M.D. 160
" at Buffalo and the West, 173
" sulphur and charcoal in, 182
" deaths of physicians by, 173
" microscopical appearances in the muscles and intestinal discharges, 173
" calomel in, by A. Hall, M.D. 219
" in Buffalo, 243
" specific reward for, 428
" Prof. Evans on spread of, 932
" infusoria in tissues and secretions, by Dr. Burnett. 552
" cases of, by Henry Y. Smith, M.D. 569
" at Buffalo in 1849, report of, by editor, 313
" Dr. Ayre's treatment of, 342, 357
" deaths by, at Boston, 376
Cyanosis, case of, by Prof. Lee, 70
Cole, Dr. W. C., on stricture of rectum, 215
Collodion, for sore nipples, 227
Coma, Anæmic, 92
Codliver oil, adulteration of, 102
" " in Phthisis, 242
" " mode of concealing taste of, 397
Calomel, influence of, on bilious secretion, 111
" in remitting fever, 415, 349
Chilblains, treatment of, 415
Carpenter's Physiology, 566
Churchill's diseases of children, 567

Colvin, Darwin, M.D. case fracture neck of femur, 285
Colegrove, C. on rupture of œsophagus, 286
Coventry, Prof. C. B., on self-reformation of medical profession, 575
Craniotomy, and child born alive, 600
Chorea, strychnia in, 600
Cretinism, statistics of, 608
Cantharadine, blistering tissue, 692
Catoræl, traumatic, case of, 705
Deafness, on, by Wm. Dufton, 118
Dysentery, Epidemic, nux vomica in, 150
Day, Dr. G. E., on dis. of advanced life, 176
Drugs, Philip Schieffelin & Co., 244
Dysentery, epidemic, by E. Stimson, M. D., 377
Dysentery, treatment of, by enemeta of tepid water, 585, 736
Delirium tremens, treated with hyosciamus, 382
Dowler, Bennet, M. D., contributions to physiology, 403, 675
Dysentery, tartar emetic in, by S. Jackson, 483
Davis, Prof. N. S., on free schools, 496
Detmold's case of abscess of the brain, 540
Doctors in Cincinnati, statistics of, 359
Delusions, Medical, history of, 716
Differences among physicians, 750
Erysipelas, nitrate of silver in, 168
" treated by congelation, 240
Erie co. poor house, report of superintendents, 424
Editorial changes, 433
Epilepsy, peculiar form of, by Prof. Hamilton, 460
Emerson on greater mortality of male children, 674
Epidemic and other diseases, notes of, by Prof. Lee, 697
Flint, Austin, M.D., on diagnosis of epidemic cholera, 121
" " lecture introductory to the principles and practice of medicine, 185
" " pleuro-pneumonitis with pericarditis, masked by delirium, 505
" " report of hospital cases, 267
" " on serous effusions within arachnoid cavity, 633
" " report on the epidemic cholera at Buffalo in 1849, 313
" " case of vomiting, with suppression of urine, 711
Fanaticism, 414
Femoral artery, ligature of, statistics, 422
Fevers, opium in, by Dr. Henry, 471
Fitch, Dr. G. N., 472
Fracture of neck of femur and autopsy, 285
Fisher, John D., obituary, 670
Fractures, remarks on, by Prof. Dugas, 727
Granger, Dr. L., on hydatids in ventricles of brain, 80
Gardner, Wm. M.D. on iodide of potassium in hydrocephalus, 138
Gunn, M., M. D., 175
Gwinelle, W. H.. M. D., casket and ribbon, 177
Glycerine, 391, 680, 334, 358
Griswold, Dr. C. D. case of western fever treated homœopathically, 463
Goddard, Paul B., M. D., on new material for anatomical injections, 493
Gooch's midwifery, 503
Gay, Martin, M. D., obituary, 557
Green, Dr. Caleb, on qualifications for studying medicine, 593
" " case of traumatic cataract, 705
Gun cotton, solution of, in Erectile tumors, by Prof. Brainard, 359
Graduates, proportion of, to population, 735
Hydrophobia, observations on, by Prof. Samuel Jackson, M. D., 30
Harris' Dental Dictionary, etc., 112
Harrison, Prof. John Pollard, decease of, 245, 493, 555
Hospital of Sisters of Charity, Buffalo, 244, 433. 310, 372
Hospital, St. Vincent's, N. Y., 431
Hospital, Massachusetts general, 680
Hamilton, Prof. F. H., report hospital cases, 65, 140, 216, 460, 568, 710
Harrison, Prof. John, memoir of, 528
Hope, James, M. D., memoir of, 82
Homœopathy and cholera, 99, 120, 547
Hardenbrook, J. K., M. D., case of tetanus, 133
Hydrocephalus, iodide of potassium in, 138
Hemorrhoids treated by nitric acid, 383, 609
" " by potassa fusa, 611
Homœopathist, trial of, for starving a patient to death, 479
Homœopathic victim, 491
Hœmorrhage nasal, 492
Hasting's practice of Surgery, 527
Horner's modified operation for excision of Os Maxillare Superius, 538
Hale, Dr. Enock, obituary, 551
Hyde, F., M. D., on Lumbar abscess, 249
Hospitals at Paris, statistics of, 301
Hospitals and Pauperism, (editorial) 303, 722
Inhalation of solid medicines, by Dr. Rodgers, 4
" of sulph. ether, death from, by Prof. Eve, 97
Illinois State Med. Association, 180
Idiots, institution for, 181
Jackson Prof. S. on tea and coffee, 161
Jarvis Dr. E. on vital force, 440
Kirk's & Paget's Physiology, 113
Kirtland, Prof. on Strychnine in malarious diseases, 289
Lee Prof. C. A. case of cyanosis, 70
" " insane in France, 514
" " absence in Europe, 376
" " notes of epidemic and other diseases, 697
Lee Robert, M. D. clinical mid-wifery, 117
Laryngitis pseudo membranous, case of, 396

2

Little George, M. D on epidemic bowel complaints, 524
Lumbar Abscess, by F. Hyde, M. D. 249
Laudanum, death by single drop of, 340
Lunatic Asylums in the United States, 354
Meningitis cerebro-spinal, by Dr. Stone, Mass., 47
Meningitis cerebro-spinal, by Dr. H. Roberts, 77
Madness extraordinary, 341
Medical appointments, 64
Malpractice prosecution for, 102, 289, 558
Meigs Prof. on obstetrics, 115
Medical practitioners, compensation for public services of, 692
Milk abscess treatment of, 368
Morphia, new preparation of, 741
Medical profession, tribute to, 183
Medicine practice of, dissertation on, 246
Mack Theophilus, M. D. on acute, idiopathic ulceration of cartilage, 385
Medical profession, self-reformation of, by Prof. Coventry, 575
Medical Colleges, statistics of, 395
Medical students in 1849, 414
Malignant tumor cured by diet of bread and milk, 419, 554
McBride Alex. M. D. case of Fallopian pregnancy, 467
McLellan Dr. Geo. obituary, 473
Medical Jurisprudence in the great desart, 477
Marriage of invalids, 481
Matico, 482
Maclise Joseph, surgical anatomy, 526
Midwifery demonstrative, 564, 621, 750
Medical profession at Paris, statistics of, 301
Midwifery Anæsthesia in, 590
Medical Journals, postage on, 632
Med. Jour. Buffalo, end of fifth Vol., 764
Medical schools, Parisian, 742, 632, 684
" " in United States, 740
Norris on ligature of femoral artery, 422
Nelligan on medicines, &c., 440
New York State Med. Society, meeting of, 630
Ozone and influenza, Dr. Spengler, 148
Ozone, its connection with epidemic diseases, 149
Ohio State Med. Convention, 151
Ohio medical plants of, Dr. Bigelow, 432
Œsophagus, rupture of, 286
Parker, W. B. case rupture of Communis Choledochus, 75
Phlebitis Portal, by Dr. Roberts, 105
Placenta, manual delivery of, 145, 369
Purpura, hæmorrhagica case of, 146
Professional appointments, 245
Pregnancy Fallopian, case of, 467
Perspiration Sanguineous, 302
Phthisis, treatment of, by Drs. Condie, and others, 601
Pharmacopœia, convention for revision of, 632
Phlebotomy in ancient times, 359
Poison, discovery of, in a body after eight years' interment, 366
Phalanx, reconstruction of, by Prof. Hamilton, 980
Professional secrets, 744
Quinia in remitting fever, by Isaac Parrish, M. D. 731
Quinia, simple mode of testing, 741
Rogers, Dr., on inhalation of solid medicines, 4, 78
Roberts, Dr., on cerebro-spinal meningitis, 77
Rectum, fissures of, by Prof. Parker, 98
Respiration, intra vaginal, by Dr. McCulloch, 104
Rheumatism, muscular and electricity, 128
Richardson, R. A., M. D., on manual delivery of placenta, 145
Rogers, Dr. S. C., case of purpura hœmorrhagica, 146
Reports, Southern Medical, 178
Rectum, stricture of, case, 215
Richardson, A., M. D., on delirium tremens, 382
Remitting fever, calomel in, 415
" " Quinia in, 731
Sherwood, Dr. W. B., on muscular rheumatism and electricity, 128
St. Louis Med. Journal, 245
Stimson, Elam, M. D., on epidemic bowel complaint, 377
Smith, W. Tyler, M. D., on paturition, &c., 440
Stanley, Edward, F. R. S,, on diseases of the bones, 502
Strychnia in malarious diseases, 289
Scarlatina, prevention of severe invasion of, 293
Scarlatina, cold water in, 363
Suicides in Paris, statistics of, 301
Smith, Henry Yale, M. D., cases of cholera, 569
Sprague, Charles, case of, (insanity,) 666
Spermatorrhœa, treatment of, by cauterization, 723
Strychnia, experiments with, on a horse, 733
Stomach, accumulation of cotton in, 741
Transactions of College of Physicians of Philadelphia, 204
Tilden & Co's medicine extracts, 500
Twins of different color, 348
Tracheotomy, case of, performed by Ricord, 360
Uric acid in the kidneys, a sign of a child having been born alive, 495
Vagina, ulceration of, connected with the states of utero-gestation and lactation, by Prof. Brainard, 729
Weed, S., M. D., on Hæmorrhoids, 383
Webster, Prof. J. W., arrested for murder of Dr. Parkman, 498
Yellow fever at New Orleans, 431
" " at Charleston, S. C., 431
Year, new, 499

See, cover, "*to readers and correspondents,*" and advertisement for *National Convention for revising the Pharmacopoeia.*

Medical Appointments. . . . . . . . . . . . . . . 64

Progress of the Cholera. . . . . . . . . . . . . . . 64

To Readers and Correspondents. . . . . . . . . . . . 64

---

*To readers and correspondents.*—The index for vol. 4, promised in our last, is in the printer's hands, but owing to the abundance of other matter for publication, is deferred until our next. Should any of the subscribers discontinue the work at the close of this volume, the index will be sent to their address, on application to the publishers.

Several communications are on file for publication. The continuation of the article by Dr. Rogers, was received too late for insertion in this No., and will appear in our next. An account of the trial of Dr. Hasbrook, may be expected in our next No.

We must again allude to the subject of typographical errors. The burthen of proof-reading falls exclusively upon us, and, with manifold other duties, we cannot devote to this task the care and attention requisite to make us independent of the indulgence of our readers and correspondents in this particular. In the report of medical cases at the Buffalo Hospital, contained in our last No., the compositor attributed to us several words, which we hope the reader did not place to our account, some of which affected the sense in a serious manner. This was owing to our being obliged to send the article to press with a single proof reading, on account of absence.

☞ The engraving of Dr. White's "Obstetrical Forceps" which accompanies this No., was accidentally omitted in the May No., and subscribers who wish to bind their volumes can have it inserted in its proper place.

